# Knowledge management in sport mega-events: A systematic literature review

**DOI:** 10.3389/fspor.2022.1056390

**Published:** 2022-12-06

**Authors:** Yuan Qin, Claudio M. Rocha, Stephen Morrow

**Affiliations:** ^1^Faculty of Health Sciences and Sport, University of Stirling, Stirling, United Kingdom; ^2^School of Social Sciences, Heriot-Watt University, Edinburgh, United Kingdom

**Keywords:** event management, knowledge transfer, Olympic Games, event legacy, sport event, major event

## Abstract

**Introduction:**

The aim of this study was to conduct a systematic review to describe and explore the current state-of-the-art of sport mega-event knowledge management research.

**Methods:**

Following the PRISMA protocol, the authors conducted a systematic search of academic and gray literature in sport, social sciences, and humanities databases. From the initial 1,751 studies reviewed, 16 met the inclusion criteria.

**Findings:**

In these studies, knowledge management in sport mega-events was mainly researched in the context of the Olympic Games. Compared with other events, the Olympic Games built a more formal knowledge management programme, which may explain why it attracted more attention.

**Discussion:**

Most of the studies highlighted the importance of tacit knowledge and individuals, as well as the needs of different stakeholder groups. Findings showed that social, cultural, political, and historical differences between hosts weaken the effect of knowledge management. Many of the published empirical studies are descriptive investigations and lack support of related theories or conceptual frameworks. The impacts of knowledge management process on the host regions and knowledge transfer between events and local stakeholders have been little explored.

## Introduction

Due to the complexity of hosting sport mega-events and the importance of event knowledge legacy, sport event managers have paid increased attention to the application of knowledge management (KM). In the literature, two main lines of thought can explain the importance of KM for effective management of sport mega-events. On the one hand, sport mega-events represent massive resource input and unprecedented challenges. Given the usual one-off nature of sport mega-events, recurring costly mistakes can and do occur (1). Many countries or cities lack the experience to host sport mega-events. Taking the Olympics Games as an example, only a handful of cities have staged the event more than once since Pierre de Coubertin reinvigorated them at the end of the 19th century (2). This is not dissimilar to other sport mega-events, such as the FIFA World Cup and Winter Olympic Games. Therefore, hosting sport mega-events requires managers not only to learn lessons and experience from previous events but also to acquire knowledge in many areas, such as catering, construction, venue management, language services, and transport management. On the other hand, sport mega-events may produce positive outcomes for host regions in relation to image, economy, tourism, and infrastructure (3). In particular, the accumulation of knowledge, such as the experience of organizing events and the skills of local staff, is an important part of the expected positive outcomes (4). The accumulation of knowledge can be an event legacy, transferable to local stakeholders to further benefit the host cities or host countries.

The formal application of KM in sport mega-events can be traced back to the 1990's. In 1998, the International Olympic Committee (IOC), in collaboration with the Sydney Organizing Committee for the Olympic Games (SOCOG), developed the Transfer of Know How (TOK) programme (5). In order to improve KM process, in 2002, the IOC formed the Olympic Games Knowledge Services (OGKS) (6). Eventually, in 2005, the IOC formally established an official KM programme, the Olympic Games Knowledge Management (OGKM) (7). Not only the Olympic Games, but also the organizers and stakeholders of other sport mega-events (e.g., FIFA and Rugby World Cup) have gradually become aware of the positive impact of KM on the development of events and host regions.

In academic research, KM in sport mega-events has attracted researchers' attention. Halbwirth and Toohey (8) introduced the concept of KM in sport mega-events associated with the 2000 Sydney Olympic Games. In this first study regarding the topic, they argued that just as other businesses, sport and event organizations need to successfully capture, share, manage and harness their corporate knowledge to reduce uncertainty of outcomes and to co-ordinate and facilitate strategy and policy implementation. From the perspective of Olympic Games, they also pointed out that every time the Games are staged there is a new Organizing Committee for Olympic Games (OCOG), and the new OCOG would face a series of issues. Examples include the cultural differences between the host destination and the previous, advances in technology, and lack of the knowledge from the past (which reflects why KM is critical for OCOGs).

But viewpoints on what KM is vary widely and have come from different disciplines, such as management, information science, and education. Davenport (9) argued that KM is the process of capturing, distributing, and effectively using knowledge. After the definition from Davenport (9), the Gartner Group defined KM as a discipline that promotes an integrated approach to identifying, capturing, evaluating, retrieving, and sharing all of an enterprise's information assets. These assets may include databases, documents, policies, procedures, and previously un-captured expertise and experience in individual workers [Gartner Group quoted in Bulter (10)]. A more succinct definition was created by Standards Australia International (11): KM refers to a multi-disciplined approach to achieving organizational objectives by making the best use of knowledge. In this paper, we propose that in the context of sport mega-events, KM can be understood as a strategy to effectively organize and host an event through a series of KM activities (knowledge identification, acquisition, creation, tailoring and storage, application, and transfer).

Despite the central importance of KM for effective management of sport mega-events, the number of studies approaching the topic remains limited. Considering that the topic has been studied for at least 20 years, this is an opportune moment to investigate the literature in a systematic way. We are unaware of any other published systematic literature review on KM in sport mega-events. A systematic review can inform researchers about gaps in the literature and need of further investigation. It can be beneficial for sport event practitioners who will have a comprehensive list of practical applications from previous studies. Therefore, the aim of this study was to conduct a systematic review to describe and explore the current state-of-the-art of sport mega-event KM research. By exploring the trends and gaps in existing research, this study can play an important role in shaping further research, policy, practice, and public perception on the topic.

## Materials and methods

**Figure 1:**
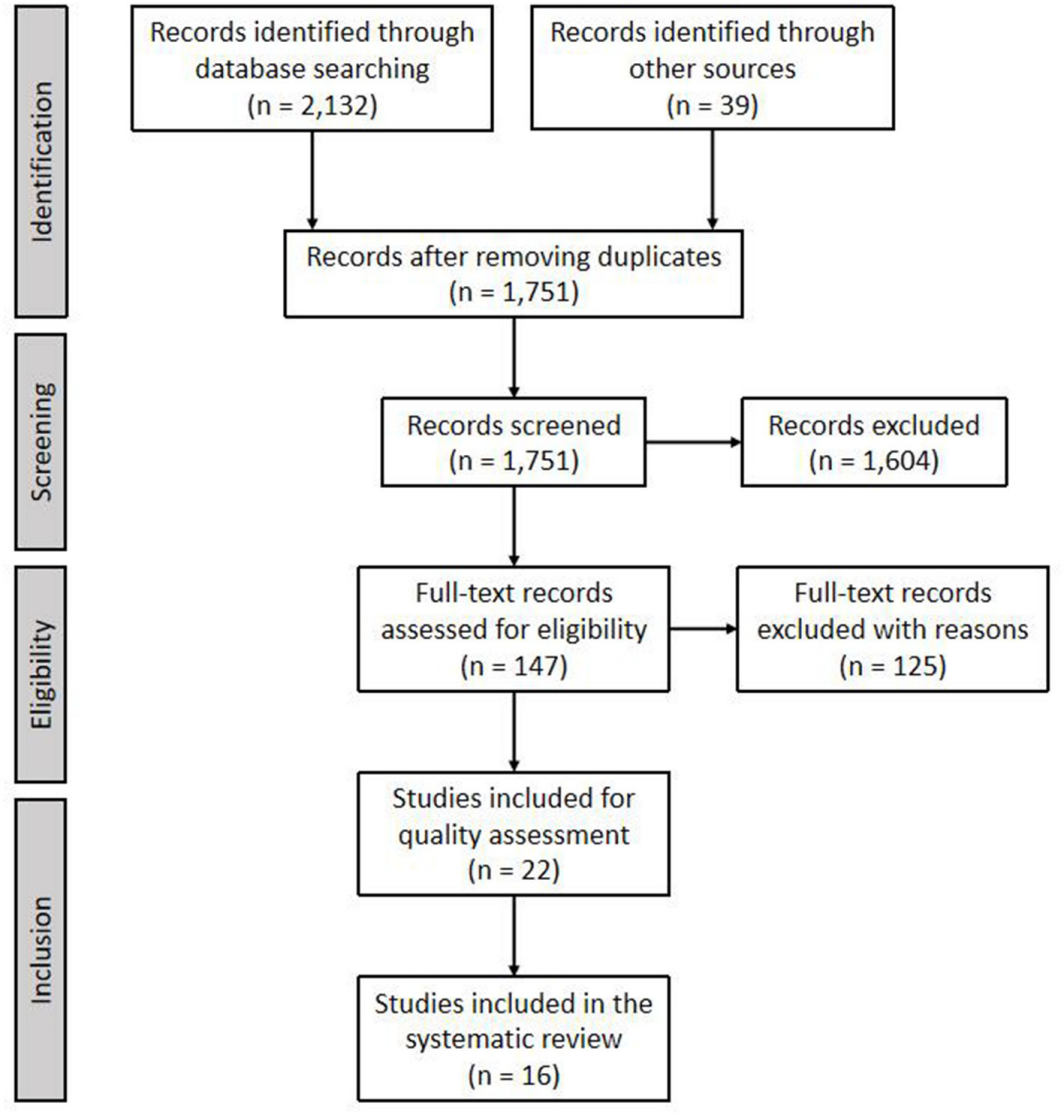
PRISMA flow diagram.

This systematic review was conducted following the PRISMA protocol (12). Figure 1 provides the PRISMA flow chart details. After listing as many words related to the topic as possible and discussing their relevance, the authors determined the search terms and relevant databases. Then, the first author conducted the initial search and downloaded all the search results, importing them into Mendeley, which allowed for duplicates to be identified and removed. Subsequent steps were study selection (including title-abstract screen and full-text screen) and quality assessment, which were undertaken independently by each one of the authors. After each step, the authors met to discuss and resolve any conflicts before moving onto the next step. Methodological details are found below.

### Search strategy

In our search, we used the Boolean operator “AND” to find studies that investigated KM in the context of sport mega-events. We used the Boolean operator “OR” to look for our terms of interest in different locations of the files, namely the title (TI), abstract (AB), keywords (KW), and subject (SU). To ensure the broadest capture of publications possible, we looked for the following search terms:

[TI (knowledge management OR knowledge transfer OR knowledge creation OR knowledge application OR knowledge storage OR knowledge identification OR knowledge acquisition OR knowledge adoption OR knowledge tailoring OR knowledge dynamics OR tacit knowledge OR explicit knowledge) OR AB (“same terms as in TI”) OR KW (“same terms as in TI”) OR SU (“same terms as in TI”)]

AND

[TI (sport^*^ event^*^ OR sport^*^ mega event^*^ OR sport^*^ major event^*^ OR Olympic^*^ OR World Cup) OR AB (“same terms as in TI”) OR KW (“same terms as in TI”) OR SU (“same terms as in TI”)].

The first author conducted a search of academic literature in sport and broader social sciences and humanities databases, specifically: WebScience, Scopus, Sport Discus, Proquest, SocIndex, Public Affairs Index, and Political Science Complete. Compared with academic literature databases, the function of advanced search of gray literature and theses databases is relatively incomplete. The above detailed search terms cannot be fully applied in gray literature and theses searching. Therefore, after discussion within the research team, the search terms used in gray literature and theses searching were simplified and only terms with high relevance to the topic were retained. The first author searched the gray literature and theses using the search string: “knowledge management” AND (“sport^*^ event^*^” OR “sport^*^ mega event^*^” OR “Olympic^*^”) on EThOS by the British Library, Explore at the British Library, Google Scholar, Open Access Theses and Dissertations (OATD) and Open Gray. The references from search results were selected according to relevance.

### Study selection

In the search, the first author downloaded all references from the search results. There were 2,171 records in total, of which 2,132 were from academic database searching and 39 from other sources. Then, the first author imported all records in to Mendeley. After eliminating duplicates, 1,751 records were found.

Next, each author independently screened the records, first with the title and abstract only and then with the full text (see Figure 1 for outcomes of the screening). Table 1 provides the pre-selected inclusion and exclusion criteria used throughout the screening process that were designed to capture as many outlets as possible. After screening with title and abstract, we excluded 1,604 records and finished with 147 full-text records to be assessed for eligibility. After reading the full text and applying the exclusion criteria, we eliminated 125 records with reasons and kept 22 records for quality assessment. Reasons for elimination are listed in the exclusion criteria (Table 1). Beyond technical analysis (e.g., dropping conceptual paper and conference abstracts), we also excluded works that discussed knowledge management but were not related to sport events and works that associated sport mega-events with education or learning but were not related to KM.

**Table 1:** Pre-selected inclusion and exclusion criteria for the systematic review.

**Inclusion criteria**	**Exclusion criteria**
Journal articles	Conceptual papers
Academic books	Conference abstracts
Academic book chapters	Works that discuss KM but are not related to sport events
Theses	Works that associate sport mega-events with education or learning but are not related to KM
Gray literature	
Empirical studies (qualitative or quantitative or mixed methods)	
Includes search term in title, abstract, keywords, or subject	
In English	

### Quality assessment

In order to assess the quality of the records for final inclusion, analysis and synthesis, the authors followed previous systematic reviews in sport management and used the American Psychology Association's guidelines (13, 14). The quality assessment resulted in 6 records being excluded due to poor research methods or lack of focus on KM, resulting in a final sample of 16 records.

### Data extraction and analysis

The following information from the final 16 records was extracted and put into an Excel table: source (journal or university), authors and date, research aim, theoretical framework, method, event, main findings, and main limitations. Each author extracted the information individually. Then, they met to discuss and resolve divergences. The analysis focused on identifying similarities and differences between the 16 records around four main points: (1) the type of events (including location and year) that have implemented KM, (2) the conceptual frameworks or theories used to study the topic, (3) the research methods, and (4) the main findings (the degree to which is applied in sport mega-events, systems or programmes used for KM, type of knowledge involved, type of stakeholders involved, and barriers in the KM process).

## Results

As shown in the Table 2, all 16 studies were articles published in peer-viewed journals. These studies were published between 2008 and 2021, with 14 of them (87.5%) published between 2011 and 2021. These articles appeared in 13 different journals, including three in Event Management and two each in European Sport Management Quarterly and Tourism Management.

**Table 2:** Summary of extracted data from studies included in the systematic review.

**Source**	**Authors (Date)**	**Research aims or questions**	**Theory/conceptual Framework**	**Method**	**Event**	**Findings**	**Limitations**
Journal of Sport and Tourism	Beesley and Chalip (6)	To examine failed efforts to transfer knowledge about non-host city leverage of the Olympic Games from Australia to Shanghai.	No theory or conceptual framework.	Semi-structured interviews (*n* = 63). Document analysis. Ethnography (observation).	Olympic Games–Beijing 2008	(1) The failure to transfer knowledge from Australian non-host regions to Shanghai about applying leverage derives from two main reasons: the difference of traditions and political systems between Australia and China; and that Shanghai was unconcerned about leveraging the Games because of the historic competition between it and Beijing. (2) Knowledge transfer must be envisioned as a far more complex task than the mere sharing of information, technology, and techniques. Rather, it requires localization with reference to social, cultural, political, and historical context.	Unique context. Single case study. Lack of theory or conceptual framework. Lack of discussion on specific measures to achieve knowledge transfer in the different socio-political context.
Event Management	Brynildsen and Parent (15)	To explore the role of test events (TE) as risk management tools by (1) evaluating how TE support the preparation and staging of the Olympic Games, and (2) assessing the feasibility of reducing their cost and scale without increasing the risks associated with hosting the Games.	No theory or conceptual framework.	Semi-structured interviews (*n* = 15). Document analysis.	Winter Olympic Games – Vancouver 2010, Sochi 2014, PyeongChang 2018	(1) Two aspects are paramount for an effective KM process: the importance of people and tacit knowledge and understanding that tacit knowledge exists through individuals. (2) TE creates opportunities to increase tacit knowledge, identify and correct gaps, and build stronger stakeholder relationships. (3) It is possible to have a lower cost/scale TE program without increased risks, though this depends on two factors: sport event hosting experience of the OCOG and having a solid and contextualized TE strategy.	Limited number of interviewees. Lack of theory or conceptual framework. The research project took place at a time (2017) when the IOC and the Olympic Games were undergoing internal governance changes as well as changes to the bid process and requirements.
Journal of Sport Management	Ellis et al. (16)	To examine impact of Olympic ambush marketing networks and knowledge transfer on the institutionalization of anti-ambush marketing legislation.	Institutional theory.	Semi-structured interviews (n = 12). Document analysis.	Olympic Games – Montreal 1976, Los Angeles 1984, Calgary 1988, Atlanta 1996, Salt Lake City 2002, and Vancouver 2010	(1) The IOC and OCOGs are highly connected within the marketing network and have greater access to information and knowledge, as well as the capacity to spread their own knowledge and opinions. (2) There were eight formal tools (the OGKM extranet, official debrief, technical manual, seminars and workshops, official observer program, final reports, research reports, and other documents) and four informal tools (OCOG presentations, other sponsor information, agencies, and Olympic gypsies) of knowledge transfer. (3) There were also several unique challenges and issues with regard to the transfer of Olympic ambush marketing knowledge, namely, trust and coordination between stakeholders, the challenges provided by the international context of the Games, and an imbalanced distribution of knowledge.	Limited number of interviewees. Lack of introduction to the cases.
Sport in Society	Frawley and Toohey (17)	To investigate how the Australian Olympic Committee (AOC) was involved in the formation of the Sports Commission (SSC) within the Sydney OCOG and as a critical contributor to the staging of the Sydney 2000.	Figurational sociological framework.	Semi-structured interviews (*n* = 22).	Olympic Games – Sydney 2000	(1) Compared with the host governments and OCOGs, the AOC seems to be better placed to leverage its prior knowledge and extensive Olympic figurations. (2) The SSC had a significant impact on the organization of sport at the Sydney Games, and the AOC had significant legal authority within SOCOG; the formation of the SSC gave the SOCOG Sport Division a very high level of importance within SOCOG.	Interview is the only source of information. There was a time lag factor associated with this study.
Geoforum	Grabher and Thiel (2)	To understand how the temporary organizations in charge of preparing a mega-event manage to mobilize the capabilities necessary for this massive venture. To examine how this enormous mobilization and concentration of resources is intertwined with the development trajectories of the projects, people, and professions.	Project Ecology theory.	Interviews (*n* = 35). Document analysis.	Olympic Games – London 2012	(1) The knowledge on preparing and performing mega-events is primarily sedimented and embodied in professionals in permanent organizations. (2) Mobilization for a large-scale temporary venture mainly happened through recruitment of qualified professionals into single-project organizations. However, this recruitment occurred mainly *via* particular channels, namely predecessor projects, personal networks, and permanent organizations in order to both increase speed and reduce uncertainty. (3) The role of permanent organizations in the context of large events is instrumental, and permanent organizations and temporary organizations complement and interact with each other.	Single case study.
Journal of Urban Affairs	Kassens-Noor (7)	To analyse the transport legacies of the Olympic Games.	No theory or conceptual framework.	In-depth interviews (*n* = 30). Document analysis.	Olympic Games – Barcelona 1992, Atlanta 1996, Sydney 2000, Athens 2004, and London 2012	(1) Even though host cities' transport systems were intrinsically different at pre-Olympics stages, similar Olympic transport systems (developed through Transfer of Knowledge program) produced similar legacies. (2) City planners can use Olympic transport features as powerful catalysts to accelerate their urban and transport plans.	Lack of theory or conceptual framework. There was a time lag factor associated with this study. Lack of introduction to the cases.
Sport Business and Management: An International Journal	Leopkey and Ellis (3)	To explore how a legacy of event hosting competencies from one event can contribute to advancing the overall hosting capacity of a nation for future events.	No theory or conceptual framework.	Document analysis.	Canada 2007 FIFA Under-20 World Cup	(1) Four broad event hosting capacity legacies from the event that potentially impacted Canada's ability to secure the FIFA Women's World Cup 2015 were identified: exemplifying success, advancement of hosting concepts, staff and leadership experience, and development and enhancement of sporting infrastructure. (2) For those in the sport event industry, the importance of hosting capacity legacies as event building blocks could mean placing a greater emphasis on the development of a national formalized, long-term, and integrated system focusing on sport event knowledge identification, acquisition, storage, and transfer.	Use of document analysis is the only source of information. Lack of theory or conceptual framework. Only two editions of FIFA-related events within one country (Canada) were considered.
Regional Studies	Mueller and Stewart (18)	To explore the significance of temporary geographical proximity for individual learning.	No theory or conceptual framework.	Questionnaires (*n* = 294).	Olympic Games – London 2012, Sochi 2014, and Rio 2016	(1) In temporary organizations and projects, knowledge retention and circulation are more dependent on individuals than on the organization. (2) When controlling for other predictors, the effect of temporary geographical proximity becomes insignificant, suggesting that it does not have a direct effect on learning. (3) The actionable relevance of the knowledge received emerges as the strongest explanatory variable for learning. (4) As sports organizations develop KM tools, it is important to address both documented and informal learning, ideally in ways that make them interact with each other.	Lack of introduction to the cases.
International Journal of Sport Management and Marketing	Parent et al. (19)	To examine the relationship between knowledge management/transfer processes and (good) governance practices in sports events.	Heisig's (20) KM activities conceptual framework.	Semi-structured interviews (*n* = 13). Document analysis.	Winter Youth Olympic Games – Lillehammer 2016	(1) Lifecycle timing is an important, but previously neglected, consideration in the sport event knowledge management/transfer process. (2) During the planning mode – the first half of the organizing committee's life – strategic-level knowledge is more important. During implementation mode, the organizing committee requires operational-level or technical knowledge. (3) While good governance might facilitate the management and transfer of knowledge, it does not ensure organizational effectiveness (performance); knowledge management/transfer processes appear more strongly linked to accountability than performance. (4) People were a more important vehicle for knowledge transfer than paper.	Limited number of interviewees. Not all interview stakeholder groups are represented in the interviews, only those key partners in the event's governance.
Sport Management Review	Parent et al. (1)	To examine the theory and practice of KM processes, using the Olympic Games as the empirical setting and the Olympic Games Organizing Committee and its stakeholders as participants.	Heisig's (20) KM activities conceptual framework. Nonaka and Takeuchi's (21) SECI model.	Semi-structured interviews (*n* = 24). Document analysis.	Winter Olympic Games – Vancouver 2010	(1) KM of Olympic Games involves the participation of internal and external stakeholders, and each stakeholder group has its own set of needs and wants. (2) The information and knowledge concepts should be placed on a continuum from explicit to tacit (with experience). (3) Socialization, externalization, combination, and internalization mechanisms can be found when tailoring knowledge for a stakeholder. (4) Knowledge sources, reasons, organizational culture, and especially individuals are important when implementing knowledge management/transfer processes.	Single case study. Lack of discussion about how the knowledge is sifted and synthesized to determine this value for a meaningful transfer.
European Sport Management Quarterly	Parent et al. (22)	To understand the government stakeholder group's coordination issues and strategies in mega-events.	Public administration theory.	Semi-structured interviews (*n* = 35). Document analysis. Ongoing notes/observations.	Winter Olympic Games – Vancouver 2010	(1) KM was one of the main issues that governments face when coordinating planning efforts for a mega-event. (2) KM issues were resolved using communication processes, decision-making frames, human resource procedures/principles, and structural frameworks.	Single case study. Lack of further exploration of the links between the various issues identified.
International Review for the Sociology of Sport	Samuel and Stubbs (4)	To explore the legacies from the greening of the Olympic Games through an analysis of Beijing 2008, Singapore 2010, and London 2012.	No theory or conceptual framework.	Semi-structured interviews (*n* = 19). Observation.	Olympic Games – Beijing 2008, Singapore 2010 (Youth), and London 2012	(1) The four determinants of green legacies are: the breadth and depth of environmental commitments during the bid process; embedding sustainability in the vision, mission, and branding of organizing committees; embedding sustainability in various aspects of Olympic organization; and knowledge transfer from one Olympic Games to the next. (2) Sustainability-related knowledge transfer is a legacy in itself. (3) Legitimation is a key strategy during the bidding process and in operations of the Olympic Games.	Lack of theory or conceptual framework. Limited number of interviewees.
European Sport Management Quarterly	Schenk et al. (5)	To examine the knowledge management and transfer (KM/KT) process within two domestic and two international sports events; to determine whether the similarities and differences between these KM/KT processes lend themselves to a single, overall sport event KM/KT process.	Heisig's (20) KM activities conceptual framework. Nonaka and Takeuchi's (21) SECI model.	Semi-structured interviews (*n* = 58). Document analysis.	2012 Ontario Summer Games, 2013 Canada Games, Glasgow 2014 Commonwealth Games, and Toronto 2015 Pan American Games	(1) The organizing committee's lifespan may influence the effectiveness of the KM/KT process and its benefit. (2) The role of individuals is important in all KM stages for domestic and international events. (3) IOC OGKM's tacit/experiential elements (e.g., observations, secondments, debriefs) are the most valuable components of that KM/KT system. (4) From a theoretical standpoint, the similar KM/KT process undertaken by event stakeholders for domestic events through Olympic-level events, regardless of the existence of a formal event KM/KT process, demonstrates the transferability potential of KM/ KT findings between event levels.	The selection of research setting is not extensive (all events were conducted in Global North countries, where access to documents may be easier due to better technological infrastructure and access to information).
Event Management	Shipway et al. (23)	To examine the extent to which the existing volunteering infrastructure supporting volunteer management in the host city were engaged before, during, and after the London 2012, to generate a volunteering legacy.	No theory or conceptual framework.	Semi-structured interviews (*n* = 16). Document analysis.	Olympic Games – London 2012	(1) Knowledge transfer processes between OCOGs and host cities seen as important but unclear, and knowledge transfer needs to take into account the volunteering culture of the host city and nation. (2) The 2012 Games raised the profile of volunteering and volunteer roles. (3) Involving existing volunteer organizations is seen as important for delivering volunteer legacies but not effectively used in London 2012. (4) Bringing forward volunteer legacy planning would have facilitated readiness for post-event social legacy initiatives to be launched immediately post-Games. In the legacy phase, existing plans should be rolled out and monitored, not conceived of at late stage.	Single case study. With the focus on volunteering infrastructure organizations, this study did not fully account for the perspectives of volunteers themselves. Lack of theory or conceptual framework.
Tourism Management	Singh and Hu (24)	To extract and organize the tacit knowledge from two organizations (Athens Organizing Committee – ATHOC, and Greek National Tourism Organization – GNTO) to discover major issues concerning the Athens 2004 Olympic Games; to identify the strategic alignment issues between the domains of Olympics planning and destination marketing and propose a conceptual framework for the future Olympic host countries.	Resource-based view theory.	In-depth interviews (*n* = 6).	Olympic Games – Athens 2004	(1) Major issues concerning 2004 Olympics: amount of investment on infrastructure and tourism services, promotional campaigns, and negative publicity in international media. (2) ATHOC was not integrated with the public sector and GNTO had no ministry of tourism until sometime before the event, which resulted in limited communication between ATHOC and GNTO. (3) There is a vast amount of tacit knowledge accumulated by key officials who are involved in organizing the mega-event and marketing the destination, and this precious knowledge source should be transferred to and re-used by future organizing committees and destination organizations again.	Limited number of interviewees.
Tourism Management	Werner et al. (25)	To explore the impact of the 2011 Rugby World Cup (RWC) on knowledge transfer (KT) processes among organizations in two regional tourism networks in New Zealand.	No theory or conceptual framework.	Semi-structured interviews (*n* = 69). Document analysis.	New Zealand 2011 Rugby World Cup	(1) The organizations in two regional tourism networks (the inter-regional tourism network and the intra-regional network) acquired valuable knowledge that may facilitate the attraction and organization of future events and enhance operational processes. (2) The most common channels of knowledge transfer operated at the firm level and included imitation/demonstration/observation, inter-firm collaboration, and document exchange. (3) Levels of knowledge transfer were higher intra-regionally than inter-regionally.	Single case study.

Regarding the type of event, 13 studies had the Olympic Games as the context, involving 10 Summer Olympic Games (Montreal 1976, Los Angeles 1984, Calgary 1988, Barcelona 1992, Atlanta 1996, Sydney 2000, Athens 2004, Beijing 2008, London 2012, and Rio 2016), four Winter Olympic Games (Salt Lake City 2002, Vancouver 2010, Sochi 2014, and PyeongChang 2018), one Summer Youth Olympic Games (Singapore 2010), and one Winter Youth Olympic Games (Lillehammer 2016). Only three studies were conducted to investigate other sport mega-events (e.g., 2011 Rugby World Cup, 2014 Commonwealth Games, and 2015 Pan American Games).

An important feature of systematic reviews is the possibility of uncovering the types of theory or conceptual framework that have informed previous studies. While half of the studies did not appear to use a theory or conceptual framework, a wide range of frameworks were used by the other 8 studies. The conceptual frameworks related to KM included Heisig's (20) KM activities framework and Nonaka and Takeuchi's (21) SECI (socialization, externalization, combination, and internalization) model. Moreover, five other theories were used once: Institutional theory, figurational sociological framework, project ecology theory, public administration theory, and resource-based view theory.

Almost all the studies used single or multiple case study strategy, applying qualitative methods to analyse data collected from interviews (*n* = 14) or documents (*n* = 12) or observations (*n* = 3). Among these studies, 12 applied more than one source of information, mainly combining interviews with observations and/or document analysis. In contrast, only one study relied on survey questionnaires to collect data, using quantitative strategies to analyse data.

With regards to the main findings, through reviewing all 16 studies, there is a clear message that KM in sport mega-events is mainly applied in the context of the Olympic Games, and only the Olympic Games and Commonwealth Games had formal KM programmes. The findings illustrate that KM in sport mega-events involves two types of knowledge: explicit knowledge (codified or written knowledge) and tacit knowledge (know-how knowledge, gained through living experience). Most of the studies emphasized the importance of tacit knowledge and the focus on individuals as the main carrier of tacit knowledge (2, 3, 5, 15, 17, 18, 24). The systematic review showed that there are many stakeholders involved in the KM process because of the complexity of sport mega-events. They are divided into internal (such as staff of organizing committees) and external stakeholders (such as the host government, media, and local community), and each stakeholder group has its own needs and wants (1, 15, 23).

Some studies examined barriers to the application of KM in sport mega-events, generally arguing that social, cultural, political, and historical differences between host destinations have become the main barrier in the KM process (6, 15, 16, 18, 22, 23, 25).

## Discussion

### Knowledge management in the Olympic Games

Among the studies found in the systematic review, 13 used the Olympic Games as the context. The key findings in these studies were: (1) the importance of TOK for the development of KM within the Olympic movement, (2) the evolution of the Olympic KM programme, (3) the initial challenges faced in implementing an official Olympic KM programme (OGKM), (4) progressing OGKM toward a more formal programme, and (5) the attempts to transfer knowledge from hosting the Games to local stakeholders.

A reason why most of the studies related to KM has been conducted in the context of Olympic Games seems to be the existence of a more formal KM programme created by the IOC–the OGKM. A formal programme provides a clear setting where KM can be investigated more easily. This fact seems to have attracted the interest of KM scholars. Another interrelated reason may be the complexity of the Olympic Games, a multi-sport event with a large number of stakeholders involved. This complexity seems to have generated needs of storing and transfer knowledge. Therefore, research and practice regarding the process of knowledge management has gained importance in the context of the Olympic Games. Only a few other events (e.g., the Commonwealth Games; the Pan-American Games) share similar characteristics, albeit in a much smaller scale.

The creation of the TOK programme represented the beginning of the KM process in the Olympic Games. In 1998, the IOC and SOCOG collaborated to develop the TOK programme. Halbwirth and Toohey (8) noted that the content of TOK was to formalize SOCOG's selling of its explicit and tacit knowledge to the IOC for $A5 million. After that, these knowledge materials would be disseminated to the OCOGs of the Salt Lake City and Athens events (8). The TOK completed both written and oral delivery of intellectual property from relevant SOCOG managers through collecting written materials and holding interviews and debriefing (27). Kassens-Noor (7) reported that through the TOK, the knowledge and experience gained from Sydney 2000 provided help for Athens 2004, specifically in terms of the transportation aspect. SOCOG laid the foundation *via* key documents on how to successfully handle Olympic transport. This allowed ATHOC to benefit from the experience of Sydney, for example, improving airports or connections between airports and the city center, creating new high-capacity transport modes, and improving additional road capacity (7).

In general, the TOK programme included both explicit knowledge transfer (written materials) and tacit knowledge transfer (interviews and debriefing). But it did not start as a KM programme, rather as an information management programme with the goal of leaving a legacy (1). After Sydney 2000, the IOC captured a huge quantity of information in the form of printed documentation, electronic files, video and audiotape through the TOK (28). However, the IOC was aware that there were still some issues, such as how they would identify the knowledge that could be transferred across time and culture to future Games organizers (28). In order to develop answers to these issues, in 2002, the IOC formed the OGKS (initially a joint venture with Monash University, but under the complete ownership of the IOC in 2004) to capitalize on the success of past events, create consistency in the delivery and management of Games, and continue a programme of knowledge capture and dissemination (6). Nevertheless, only one of the selected studies (6) mentioned the OGKS.

In 2005, the IOC integrated the business process of the OGKS and evolved it into the OGKM, a formal KM programme focusing on Olympic knowledge transfer. However, there were some challenges at the early stage of the OGKM. For example, Sydney 2000 successfully played the role of leveraging–a strategic approach to event management to maximize economic, social, and environmental gains to a destination before, during, and after an event–which not only promoted the economic and social development of the host destination but also benefited the non-host region, especially the Hunter Valley region (6). While the IOC desired to see the lessons learned through the Sydney Olympics carried forward, Greece was unable to fully realize the benefits in the lead-up to the 2004 Olympic Games (6). Similarly, in the context of Beijing 2008, there was a failure to transfer knowledge regarding leverage of the Olympic Games on non-host regions from Australia to Shanghai. Beesley and Chalip (6) pointed out that the main reason for this failure was that the social, cultural, and political conditions in China and Shanghai made the Australian knowledge incompatible with local systems, values, and tourism development vision.

After 2008, the IOC placed greater apparent emphasis on the implementation of OGKM. The Vancouver 2010 Winter Olympic Games was the first to see a more formal OGKM in place, starting at the bid stage. The Vancouver Olympic Winter Games Organizing Committee (VANOC) built an intranet (ICE) and an extranet (SNOW), allowing knowledge sharing for all who had access (1). These VANOC intranet/extranet combined with the OGKM extranet housed the information and knowledge produced, such as technical manuals, meeting minutes, policy documents, daily reports, and final reports (1). VANOC also actively fulfilled the requirements of OGKM programme for the OGs, such as hosting secondees and observers from Sochi (the next Winter Games host) before and during the Games, conducting a debrief post-Games, and submitting material for the OGKM extranet for future hosts to examine (1). An example on how OGKM became more formal in Vancouver 2010 can be found in the transfer of post-Games legacy to London 2012 and future Games. As part of its sustainability legacy for future organizing committees, VANOC created a new sustainability governance model for organizations staging mega-sporting events, which included reporting frameworks and a sustainable sport event toolkit (4). There were also five annual sustainability reports produced by VANOC, which covered what was promised, what VANOC was able to control, and finally how VANOC performed against its goals (4).

London 2012 was the most investigated event, with five studies, but only two studies explicitly investigated the application of KM (2, 23). Unlike other selected Olympic studies which mainly focused on knowledge transfer from the host destination to future hosts, these two studies explored knowledge transfer from the Games to local stakeholders. Grabher and Thiel (2) noted that in order to help accomplish planning and organization of large-scale events such as the Olympic Games, the huge temporary organizations setup exclusively for the events have to mobilize knowledge and capacities from professionals. They found that this process is intertwined with three different learning trajectories (projects, people and professions). In particular, the learning trajectory of professions include a knowledge transfer from professional staff to local industry. From the perspective of construction industry, Grabher and Thiel (2) pointed out that the learning trajectories extended beyond the end of the construction for the Games. Through the continuation of professionals' post-Olympic careers in other organizations, the experience gained was disseminated into the next generation of large-scale construction ventures in the UK (2). Shipway et al. (23) studied the extent to which there was engagement with the established volunteering infrastructure in the host city to achieve positive legacy outcomes. They mentioned that there was a knowledge transfer between the IOC and London's volunteering infrastructure. While this study did not explain how this knowledge transfer was applied in the process of promoting volunteer legacy, it pointed out the shortcomings of this knowledge transfer (non-transparency of the mechanisms for KM and lack of consideration for local volunteering culture).

Although the selected studies involved 16 Olympic Games, most of the Games were not studied in detail. The Olympic Games before 2000 received less attention because they did not have much connection with the KM process. Some studies did refer to knowledge accumulated which was involved in subsequent knowledge transfer (e.g., transport legacy of Barcelona 1992 and Atlanta 1996) (7, 16). In addition, some studies focused on multiple Olympic Games, but the KM of Olympic Games was just a part or a factor in their topics. In such studies (e.g., 17, 19), the authors did not specifically explore how the selected Games participated in the KM process. For instance, Brynildsen and Parent (15) investigate how test events enhance the knowledge of hosts regarding risk management.

### Beyond the Olympics: Knowledge management in other sport-mega events

Three studies explored KM in the context of other sport mega-events, each focusing on different topics and providing different key findings. These studies explored (1) the contribution of event knowledge legacy in advancing a nation's sport event hosting capacity, (1) the influence of knowledge transfer on the host region's tourism network, and (3) other international events' attempts and efforts to build formal KM programmes. The studies paid more attention the impact of KM on the host regions, which is different from the studies conducted in the context of Olympic Games.

Leopkey and Ellis (3) investigated how the event hosting capacity legacies from the Men's Under-20 2007 FIFA event contributed toward winning the rights to host the Women's FIFA World Cup 2015 event. To explain the research question, Leopkey and Ellis (3) utilized Chappelet's (26) sport event hosting strategy. Chappelet (26) noted that some cities have sought to host smaller scale events in order to acquire the relevant skills and experience needed to host more complex events. This process includes a knowledge transfer from smaller events to local event organizers or staff. Leopkey and Ellis (3) argued that hosting the 2007 event provided the opportunity for event workers to develop their event experience, knowledge, and leadership skills. The staff and leadership experience form the 2007 event can be regarded as an event knowledge legacy. Leopkey and Ellis (3) reported that locally, some staff and volunteers with experience and knowledge from the 2007 event continued to advance and play more significant roles in future FIFA events and sport venues management in the country. For instance, Valerie Hughes, General Manager at the Women's World Cup 2015 venue in Ottawa, gained experience from working as the National Event Manager at the 2007 event.

Werner et al. (25) explored the impact of the 2011 Rugby World Cup on knowledge transfer processes among organizations in two regional tourism networks in New Zealand. They found that during the event, a large amount of new knowledge from international corporations, sporting bodies and international sponsors, as well as new personnel with international events experience, was brought into the host destination. Through knowledge transfer processes, the member organizations from two regional tourism networks (the inter-regional tourism network and the intra-regional network) both acquired valuable knowledge that may facilitate the attraction and organization of future events and enhance operational processes (25).

Schenk et al. (5) explored the commonality of sport event knowledge management and transfer (KM/KT) process through examining KM/KT process within two domestic and two international sports events (2014 Commonwealth Games–CWG–and 2015 Pan-American Games–PAG), introducing the application of KM in CWG and PAG, respectively. In the context of the CWG, the Commonwealth Games Federation (CGF) is the event's rights holder. The CGF has used the Event Knowledge Services (EKS) to create, transfer and manage knowledge (5). Like the OGKM, the EKS represents a specific setting where KM can be investigated. However, other events that still do not count with a formal KM programme have also attracted interest. For example, the PAG did not have a formal KM/KT system in place, but KM has been investigated in the context of the PAG (5). The complexity of this multi-sport mega event (and even the structural similarity with the Olympic Games) seems to explain the research interest in the PAG and even in other smaller events (5).

### Formal knowledge management programmes

Among the mega-events in the selected studies, only the Olympic Games and Commonwealth Games had formal KM programmes. The OGKM, a formal KM programme of the Olympic Games establishing in 2005, has been investigated (1, 5, 7, 16, 18, 19). The OGKM consists of three main elements: consultation services, personal experience, and information transfer (1, 29). Kassens-Noor (7) reported that the IOC consultation services cover multiple seminars and workshops: the applicant and candidate city seminars (before the host city selection); the foundation seminar for the selected host city; the observer programme for future hosts during the preceding Games; the debrief sessions with the recent Olympic host post-Olympics, and topical experts for offer advice. The personal experience element allows core staff of future Games to actively participate in preceding Games. It includes the formal observer programme (future hosts visit current hosts) and the secondee program (staff from future Games fill short-term positions at current Games) (7). The information transfer element is an IOC-required documentation process of Olympic planning and execution with more than 90 technical manuals, films and photos, other knowledge reports, and the Official Games Report post-Olympics. All knowledge is accumulated in the OGKM Extranet (7).

The KM programme of CWG was just mentioned by Schenk et al. (5). There is little information about this programme. Schenk et al. (5) reported that 2014 CWG had a formal KM programme and retained a commercial organization (EKS) to manage its KM programme. Compared with the Olympic Games, it seems that CWG has not internalized its KM programme yet.

### Definitions and types of knowledge

The key findings of studies in this theme are related to the general acceptance of two types of knowledge–explicit and tacit–and the importance of tacit knowledge possessed by individuals.

Different definitions of knowledge, particularly the relationship between information and knowledge, were utilized in the selected studies. While Beesley and Chalip (6) and Werner et al. (25) defined knowledge as information with meaning that exists within the individual, Parent et al. (19) argued that knowledge is more than information (know-what), it is also know-how and constitutes one of the most valuable organizational assets. Parent et al. (1) also found that the concepts of information and knowledge should be viewed as a continuum rather than as a knowledge hierarchy. However, within some of the studies included in the final sample of this systematic review, two types of knowledge are usually defined: tacit knowledge (i.e., know how) and explicit knowledge (i.e., know what) (30). In the context of sport mega- events, explicit knowledge is more easily articulated, written or codified (30); it can be translated into words or symbols, and thus be transformed into technical books, films, or official knowledge reports of sport mega-events. On the contrary, tacit knowledge (e.g., expertise, staff experience, and personal skills) is more difficult to translate and to explain to outsiders (25). Tacit knowledge can sometimes only be learned through practice and direct contact with the person who possesses it (31).

Most of the studies highlighted the importance of tacit knowledge. Brynildsen and Parent (15) and Schenk et al. (5) both noted that there are two paramount aspects for an effective KM process and a successful preparation of the event: valuing the importance of tacit knowledge, understanding that tacit knowledge exists through individuals. In the pre-event stage, planning and organizing sport mega-events are accomplished by specialized project organizations (e.g., OCOG) (2). Due to their inherently temporary nature, these organizations cannot provide the specialized knowledge and specific “project capabilities” on their own, but have to mobilize the knowledge from the past and from outside (2).

Hence, knowledge in such organizations is mostly embodied in individuals who provide diverse expertise and skills (2, 17, 18). Furthermore, in the post-event stage, the role of individuals in the transfer of event knowledge legacy (e.g., staff skills and leadership experience), has also been crucial. Parent et al. (1) argued that in the context of the Olympic Games, the transient workers or staff with previous experience working with OCOGs are called “Games gypsies.” The tacit knowledge of Games gypsies is a valuable asset to organizing committees. Since Sydney 2000, OCOGs have kept a focus on those who hold the most valuable tacit knowledge to ensure their skills or experience can be best transferred to the next event and benefit the hosts (3, 5, 24). This strategy has been effective to transfer knowledge from one host to the next. However, the strategy also illustrates how very little attention has been paid to knowledge transfer from the organizing committees to local communities. The lack of consideration for KM to the local area is an important finding of the current review, mainly if we consider that sport mega-events have been majorly funded with public funds.

### Stakeholders in knowledge management process

Most of studies in the systematic review highlighted that different stakeholders are involved in the KM process (1, 3–5, 15–17, 19, 22, 23). Even though among these studies there are slight differences in the views on the composition of stakeholders, it can be generally concluded that sport mega-event KM process mainly involve internal stakeholders and external stakeholders. The internal stakeholders mainly refer to the organizing committee's staff and volunteers. The external stakeholders include the host (local/regional and national) governments, the media (print, radio, photography, broadcasting and social media), national and international sponsors, the international delegations (athletes, coaches, support staff, etc.), regional/national and continental/international sport federations, and the community (schools, residents, activists, local business and tourism organizations, etc.). In addition, each stakeholder group has its own set of needs and wants, and there can be heterogeneity of these needs/wants within a given stakeholder group (1, 15, 23).

### Barriers for knowledge management

The barriers in KM process of sport mega-events were also raised by researchers [e.g., (6, 15, 16, 18, 22, 23, 25)]. We found that three barriers have been identified: trust and coordination between stakeholders, an imbalanced distribution of knowledge, and the context differences between host destinations.

Schenk et al. (5) investigated the KM process of the 2014 CWG and 2015 PAG. They found that some stakeholders did not either have access to the event's KM program or see the need to access it. This demonstrated that there may be barriers either in knowledge sharing or in communication and trust between stakeholders (5). Ellis et al. (16) found that despite the increased time and effort involved in the OGKM programme, knowledge transfer to stakeholders other than OCOGs was still limited. It led to an imbalanced distribution of knowledge (16).

It is worth noting that seven studies in the review highlighted the context differences between host destinations. Beesley and Chalip (6) found that the failed efforts to transfer knowledge about non-host city leverage of the Olympic Games from Australia to Shanghai was mainly caused by political differences between China and Australia. In the context of London 2012, Shipway et al. (23) examined the knowledge transfer between the IOC and London's volunteering infrastructure. They noted that knowledge transfer needs to take into account the volunteering culture of the host city and nation. Brynildsen and Parent (15) also pointed out that different understanding and perception due to language and culture is an issue in the KM process. Werner et al. (25) concluded that knowledge transfer is a far more complex task than the mere sharing of information and techniques. Rather, knowledge transfer requires localization with reference to social, cultural, political, and historical context. Successful knowledge transfer requires an understanding of the ways that an event, such as the Olympic Games, is framed and understood by its host (6). Therefore, to a great degree, in the context of sport mega-events, social, cultural, political, and history differences between host destinations have become the main barrier in the KM process.

### Conceptual frameworks

Regarding conceptual frameworks, two main findings were identified in the review: the frequent use of two KM conceptual frameworks, and the adoption of a limited number of theories to discuss KM.

In the selected studies, the conceptual frameworks related to describe the creation and transfer of knowledge were utilized most (*n* = 5). These frameworks include Heisig's (20) KM activities framework and Nonaka and Takeuchi's (21) SECI model. Heisig's (20) framework refers to the most frequently discussed activities in the KM process: knowledge acquisition (collecting/harnessing knowledge needed to undertake a certain task), application (using knowledge needed to perform a given task), creation (producing new knowledge), identification (ascertaining the knowledge required to undertake a given task), storage (retaining, protecting and maintaining knowledge useful for a given task for subsequent use), and transfer (knowledge delivery from sender to receiver). In the systematic search of the current study, we include these six activities as search terms.

Nonaka and Takeuchi's (21) SECI model is concerned with knowledge creation and involves the transfer of both tacit and explicit knowledge between individual, group, and organization levels, which interact in a “knowledge spiral” resulting in knowledge creation. This model divides knowledge creation processes into four categories: socialization (tacit knowledge formation and communication), externalization (formation of explicit knowledge from tacit knowledge), combination (use of explicit knowledge), and internalization (formation of new tacit knowledge from explicit knowledge).

Other studies in the final collection drew upon different theories, such as institutional theory, and figurational sociological framework (see Table 2). The studies using these theories have similar characteristics that they explored the relationship between their main research objects and KM, or their research content involved KM.

The basis of institutional theory is that organizations adopt practices and structures based on social expectations of specific norms, beliefs, and values determined by institutions in their external environment (32). Among the selected studies in this systematic review, only Ellis et al. (16) drew upon institutional theory to investigate how the transfer of ambush marketing knowledge have influenced institutional rules toward anti-ambush practices. They found that many consultant agencies have help to foster new institutional rules to prevent ambush marketing *via* knowledge transfer, usually from former IOC and OCOG members to host cities.

Frawley and Toohey (17) used a figurational sociological framework to explore the interdependent and changing relationships between the Australian Olympic Committee (AOC), the SOCOG, and the New South Wales Government. In this framework, figurations are conceptualized as structures of mutually oriented and dependent people who operate within historically produced, interdependent networks. Figurations, such as those between organizations, are also viewed as dynamic, or “in process”, because power relations between individuals and/or groups are continually in flux, rather than being static or still. Frawley and Toohey (17) found that the AOC can gain a strategic advantage over the other Australian Olympic stakeholders, in a large degree because of its prior Olympic knowledge.

We see a limited use of theories in the study of KM in sport mega-events. There are possibilities for expanding the application of theories that have been used before. For instance, the use of institutional theory was used only to explain effects on the structure of marketing networks. However, this theory can be applied to investigate and explain some limitations of the current approach KM programmes. The OGKM has promoted an isomorphic way to transfer knowledge between host destinations. This way may not be the most effective one taking into consideration the socio-economic-cultural differences among host destinations. Studies drawing upon other theories are still missing. The absence of studies drawing upon stakeholder theory is likely to be the most striking one. Stakeholder theory stresses the interconnected relationships between an organization and those who have a stake in it (e.g., customers, suppliers, employees, communities, and others) (33). Whilst some scholars propose that the interests of all stakeholders have intrinsic value (34), others assume a more pragmatic view of the stakeholder theory, defining who really matters as a stakeholder for specific organizations (35). Considering that the current state-of-the-art of KM literature shows the fundamental role of different stakeholders for tacit knowledge transfer, the literature still lacks a deeper analysis about the role of different stakeholders in the process.

## Conclusion

This systematic review has demonstrated the state-of-the-art of KM in sport mega-events. Although more and more practitioners and academics have become aware of the positive role of KM, the application of the concept is still limited and focused mainly on the Olympic Games. While a few other events have sought to investigate or apply KM, such events still do not have a formal KM programme or system in place. Furthermore, it is important that KM in sport mega-events should focus on tacit knowledge and individuals (the important carrier of tacit knowledge), as well as the needs of different stakeholder groups. For organizers and managers of sport mega-events, KM is a much more complex task than just receiving and transferring information and techniques. Successful KM requires a comprehensive understanding of the host destinations' social, cultural, political, and historical context. Knowledge needs to be tailored and adapted to make it useful in the context to which it is to be applied.

The systematic review revealed some limitations in the published empirical studies and possibilities for future studies. Fifty per cent of the selected studies were conducted as descriptive investigations, with no theory or conceptual framework supporting the research. Even though these descriptive studies have implications to the field of KM in sport mega-events, it is desirable that more theory-based studies are conducted. One possibility for future studies is to expand the use of conceptual frameworks related to KM and stakeholder theory. In particular, stakeholder theory deserves more attention. Due to the complexity of organizing sports mega-events, the KM process involves the participation of many different stakeholders, which are fundamental for tacit knowledge transfer, which in turn is a central element in KM. Moreover, most of the studies tend to investigate knowledge transfer between host destinations or organizing committees to the future hosts. But knowledge transfer between event and local stakeholders or impacts of KM process on host regions was explored by few studies. Benefiting the host region is one of the main purposes of applying KM in sport mega-events, and this topic is worth receiving more attention. We suggest that future studies can focus more on how KM in sport mega-events influences local stakeholders, such as local governments, sport federations, and communities.

## Data availability statement

The original contributions presented in the study are included in the article, further inquiries can be directed to the corresponding author.

## Author contributions

All authors listed have made a substantial, direct, and intellectual contribution to the work and approved it for publication.

## Funding

This work was supported by the University of Stirling Open Access and Article Processing Charge Fund.

## Conflict of interest

The authors declare that the research was conducted in the absence of any commercial or financial relationships that could be construed as a potential conflict of interest.

## Publisher's note

All claims expressed in this article are solely those of the authors and do not necessarily represent those of their affiliated organizations, or those of the publisher, the editors and the reviewers. Any product that may be evaluated in this article, or claim that may be made by its manufacturer, is not guaranteed or endorsed by the publisher.
